# Magnetic levitation pump versus constrained vortex pump: a pilot study on the hemolysis effect during minimal invasive extracorporeal circulation

**DOI:** 10.1186/s13019-021-01637-1

**Published:** 2021-09-08

**Authors:** Ignazio Condello, Giuseppe Santarpino, Giuseppe Filiberto Serraino, Pasquale Mastroroberto, Giuseppe Speziale, Giuseppe Nasso

**Affiliations:** 1 Department of Cardiac Surgery, GVM Care & Research, Perfusion Service, Anthea Hospital, Via Camillo Rosalba 35/37, 70124 Bari, Italy; 2 Department of Cardiac Surgery, Paracelsus Medical University, Nuremberg, Germany; 3 Cardiac Surgery Unit, Department of Experimental and Clinical Medicine, University “Magna Graecia” of Catanzaro, Catanzaro, Italy

**Keywords:** Cardiopulmonary bypass, Hemolysis, Magnetic levitation pump, Constrained vortex pump, Minimally invasive extracorporeal circulation, Coronary artery bypass grafting

## Abstract

**Background:**

Elevated plasma free hemoglobin is associated with multi-organ injury. In this context, minimally invasive extracorporeal technologies represent a way to reduce this complication following cardiac surgery.

**Methods:**

We present a pilot study focused on plasma free hemoglobin levels in 40 patients undergoing isolated coronary artery bypass grafting (CABG). The same circuits for minimally invasive extracorporeal circulation (MiECC) were used in all patients. The ECMOLIFE magnetic levitation pump was used in the study group (n = 20), and the AP40 Affinity CP centrifugal blood pump was used in the control group (n = 20).

**Results:**

In the immediate postoperative period, plasma free hemoglobin (PFH) and lactate dehydrogenase (LDH) were significantly lower in the study group than in the control group (10.6 ± 0.7 vs 19.9 ± 0.3 mg/dL, *p* = 0.034; and 99.16 ± 1.7 vs 139.17 ± 1.5 IU/L, *p* = 0.027, respectively). Moreover, patients treated with the magnetic levitation pump showed lower creatinine and indirect bilirubin (0.92 vs 1.29 mg/dL, *p* = 0.030 and 0.6 ± 0.4 vs 1.5 ± 0.9 mg/dL, *p* = 0.022, respectively) at 24 h after the procedure, and received fewer transfusions during the whole postoperative period (3 vs 9 red blood cell units (RBC), *p* = 0.017).

**Conclusion:**

Our pilot study suggests that the use of magnetically levitated centrifugal pumps for extracorporeal circulation support is associated with a lower risk of hemolysis, though larger studies are warranted to confirm our results.

## Introduction

Minimizing the risk of blood damage (i.e. hemolysis) using minimally invasive extracorporeal technologies is critical, especially if patients show higher hematocrit values during cardiopulmonary bypass (CPB).

The recent generation constrained vortex pumps, with their inherent design improvements, could lead to a reduction in red blood cell trauma. However, this topic is a source of potential bias, including the use of magnetically levitated pumps [1, 2]. In particular, hemolysis and plasma free hemoglobin release during extracorporeal circulation can occur from a number of patient-related and technical factors and might be worse with high flows and/or excess negative pressures within the circuit and blood transfusions [1, 2]. Elevated plasma free hemoglobin is associated with multi-organ injury, including severe acute kidney injury [3].

In this context, we present a pilot study on two different pump technologies, a magnetic levitation pump vs a constrained vortex pump, comparing 40 patients undergoing isolated coronary artery bypass grafting (CABG) using a minimally invasive extracorporeal circulation (MiECC) type IV system, which enables volume management.

## Materials and methods

Between September 2019 and September 2020, 40 consecutive elective patients undergoing isolated CABG with MiECC type IV were included in the study.

The study period and the patients to be enrolled were selected on the basis of the number of magnetic levitation pumps to be tested and a request was made for comparing them in a pilot study with the centrifugal pumps normally used in our center. The study protocol was approved by the local ethics committee and informed consent was obtained from all individual participants included in the study. Patients with chronic kidney disease, type 1 or 2 diabetes mellitus, anemia or other individual risk factors for hemolysis were excluded. The decision to perform CABG with MiECC was left to the cardiac surgeon in accordance with the perfusionists team. During the study period, 196 isolated CABG operations were performed; of these, 156 were excluded based on the afore mentioned exclusion criteria and 40 patients consented to participate in the study.

Modular MiECC type IV with the ECMOLIFE magnetic levitation pump was used in the study group (Levitation Group, n = 20), and modular MiECC type IV with the AP40 Affinity CP centrifugal blood pump was used in the control group (Vortex Group, n = 20).

Closed circuit was performed with modular MiECC, whose design presents the characteristics of a volume management circuit (MiECTiS classification). A shunted venous soft-shell reservoir was used, the aortic root and pulmonary artery suction was managed in series venous return. The reference value for the management of venous drainage was the central venous pressure, maintained using urapidil as a vasodilator for higher values, or upon request of drainage by the surgeon, using the Trendelenburg position for lower values. All patients were treated with mild hypothermic CPB (34 °C to 36 °C).

All CABG procedures were performed under cardioplegic arrest through median sternotomy and oro-tracheal intubation. During surgery and postoperatively, the need for blood transfusion was established according to the institutional protocol of the heart team based on the patient’s oxygenation status in addition to predefined hemoglobin levels. In any case, blood volume was never re-transfused from the cell saver.

**Fig. 1:**
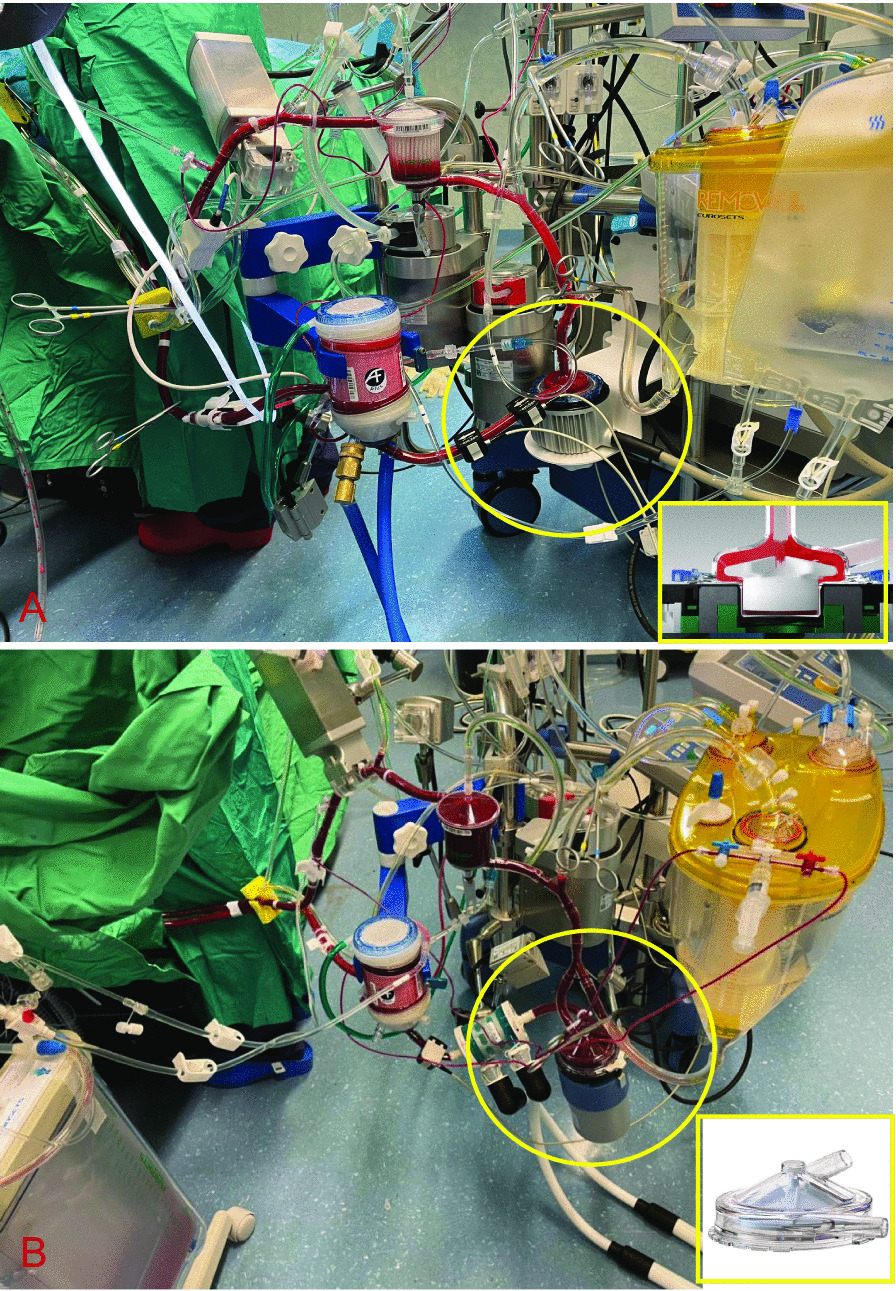
**A** MiECC with magnetic levitation pump, ECMOLIFE (highlighted); **B** MiECC with constrained vortex pump, AP40 (highlighted)

Normothermic CPB was instituted with aortic and double-staged venous cannulas. Two types of heart lung machines were used:

- In the Levitation Group, the ECMOLIFE console (Eurosets, Medolla, Italy) with the ECMOLIFE centrifugal pump (Eurosets, Medolla, Italy) was used (Fig. 1A);
- In the Vortex Group, Stockert S5 (cHLM) with Bio-console 560 (Medtronic Bio-Medicus, Inc., Eden Prairie, MN) with the AP40 Affinity CP centrifugal pump (Medtronic Bio-Medicus, Inc., Eden Prairie, MN, USA) was used (Fig. 1B).

In both groups, the following components were used for the MiECC type IV circuit (Fig. 1):

- Oxygenator alone (Eurosets, Medolla, Italy);
- Venous bubble trap (Eurosets, Medolla, Italy);
- Soft shell venous reservoir (Eurosets, Medolla, Italy);
- Landing monitoring systems (Eurosets, Medolla, Italy).

In both groups, the MiECC procedure was managed without using extra-cavitary suction but using a cell saver [4].

Static priming volume mean values (mv): 450 ± 35 mL; Circuit length (mv): 1 m^2^; no retrograde autologous priming was made.

Normothermic blood cardioplegia (St. Thomas solution) was used in all cases and repeated every 20 min.

During CPB, the following parameters were measured and collected (every 5 min) in both groups:

- Cardiac index,
- Indexed oxygen delivery,
- Central venous pressure,
- Inlet pressure of the centrifugal pump,
- Revolution per minute of the pump and the oxygenator pressure drop,
- Pressure of arterial cannula,
- Hematocrit,
- Hemoglobin.

After CPB, the following parameters were measured in both groups:

- Plasma free hemoglobin (10 min after CPB),
- Lactate dehydrogenase (at 24 h),
- Indirect bilirubin (at 48 h),
- Creatinine (postoperative peak),
- Total red blood cell units administered intra- and postoperatively.

## Results

The patient characteristics and the perioperative results are described in Table 1.

**Table 1:** Patient characteristics intra and postoperative results

	Levitation Group (n = 20)	Vortex Group (n = 20)	*P*-value
*Preoperative*			
Age (years) (mean ± SD)	71.0 (63.7)	69 (58.7)	0.89
Body surface area (m^2^)	1.83	1.82	0.94
EuroSCORE II	1.5	1.7	0.88
Pre-CPB hematocrit (%) (mean ± SEM)	34.6 ± 1.3	34.8 ± 2.1	0.99
Hb (g/dL)	12.3 ± 1.1	12.3 ± 1.2	1
Serum creatinine (g/dL)	0.83 ± 0.5	0.85 ± 0.7	0.96
PFH (mg/L)	0.02	0.01	1
*Intraoperative*			
CPB time (min)	72 ± 15.2	71 ± 7.36	1
Aortic cross-clamp time (min)	52 ± 9	51 ± 7	0.93
DOi_2_ (mL/min/m^2^)	339 ± 20	338 ± 17	0.99
CI (L/min/m^2^)	2.4 ± 0.2	2.4 ± 0.1	0.99
Pump speed (rpm)	2800 ± 140	2880 ± 160	0.98
Pump inlet pressure (mmHg)	56 ± 10	58 ± 9	0.88
CVP (mmHg)	5 ± 3	6 ± 2	0.89
Hct (%)	34 ± 2	32 ± 1	0.69
Hb (g/dL)	11.5 ± 0.5	11.3 ± 0.6	0.76
Oxygenator pressure drop (mmHg)	45 ± 4	41 ± 2	0.79
Arterial pressure cannula (mmHg)	105 ± 9	104 ± 7	0.89
MAP (mmHg)	65 ± 7	63 ± 4	0.91
*Postoperative*			
PFH at 10 min after CPB (mg/L)	10.6 ± 0.7	19.9 ± 0.3	0.034
LDH at 24 h after CPB (IU/L)	99.16 ± 1.7	139.17 ± 1.5	0.027
Creatinine peak after CPB (mg/dL)	0.92	1.29	0.030
RBC (units)	3 (0.15/patient)	9 (0.45/patient)	0.017
Indirect bilirubin after CPB (mg/dL)	0.6 ± 0.4	1.5 ± 0.9	0.022

CI, cardiac index; CPB, cardiopulmonary bypass; CVP, central venous pressure; DOi_2_, indexed oxygen delivery; Hb, hemoglobin; Hct, hematocrit; LDH, lactate dehydrogenase; MAP, mean arterial pressure; PFH, plasm free hemoglobin; RBC, red blood cells; SD, standard deviation; SEM, standard error of the mean

No deaths or major postoperative complications were recorded in the 40 patients in the postoperative period.

## Discussion

Our pilot study suggests that the use of magnetic levitation pumps can reduce the degree of hemolysis in patients undergoing CABG with modular MiECC type IV, though confirmation from larger studies is required.

It was demonstrated that free-hemoglobin is not significantly increased by centrifugal pump [5]; however, no study reported a difference between the levitation pump and the centrifugal pump on this hemolysis endpoint.

To the best of our knowledge, this is the first study using this MiECC system in this patient subset. The promising results recorded may provide direction for further research on this topic, which may have an impact on postoperative clinical outcomes as partly observed in our pilot study.

The major limitation of our study is the small sample size, as only 20 magnetic levitation centrifugal pumps were received or tested. That is the reason for the study design: this is not a randomized study but only a pilot study aiming to assess, as first endpoint, the safety and feasibility of this new technology. Another limitation is the lack of haptoglobin measurement; plasma free hemoglobin and lactate dehydrogenase were used as hemolytic indices.

Moreover, the higher number of transfusions in the Vortex Group can represent a bias for the increased postoperative bilirubin and lactate dehydrogenase. However, this may support the advantage of using the magnetic levitation pump.

The clinical significance of our study can easily be understood: current guidelines suggest that MiECC systems should be used routinely, especially in the field of coronary surgery [6]. Furthermore, recent studies [7, 8] suggest to improve minimally invasive extracorporeal systems towards minimal biological invasiveness and greater biocompatibility. The use of MiECC with a magnetic levitation centrifugal pump, if it confirms its ability to reduce hemolysis on larger scale samples, could represent an important technological advance in this direction.

## Data Availability

The datasets used and analysed during the current study are available from the corresponding author on reasonable request.

## Abbreviations

MiECC: Minimal invasive extracorporeal circulation
DO2i: Indexed oxygen delivery
O2ERi: Indexed oxygen extraction ratio
CI: Cardiac index
CPB: Cardiopulmonary bypass
CVP: Central venous pressure
Hb: Hemoglobin
Hct: Hematocrit
LDH: Lactate dehydrogenase
MAP: Mean arterial pressure
PFH: Plasm free hemoglobin
RBC: Red blood cells
SD: Standard deviation
SEM: Standard error of the mean
